# Treatment of the Hemorrhagic Diathesis

**Published:** 1843-01

**Authors:** 


*Treatment of the Hemorrhagic Diathesis.—The London and Edinburgh Monthly Journal of Medical Science* for July last contains an exceedingly interesting paper on this subject, read before the Medico-Chirurgical Society of Edinburgh, by James Miller, the successor of Sir Charles Bell, in the Surgical chair of the University of Edinburgh.

To constitute the hemorrhagic diathesis, we have not only, Mr. Miller observes, the blood flowing through dilated and non-contractile tubes, but sent thither in greater volume than in ordinary and healthy circumstances, thinner and more fluent than in health, and little if at all able to arrest its own course by assuming the solid form. In addition, the capillary tunics are not only thin, but weak, and easily lacerable; a slight bruise produces serious ecchymosis; coughing may induce hæmoptysis; a sneeze brings on epistaxis; diarrhœa occasions copious evacuations of blood by the rectum; and extravasations are not unlikely to follow but slight causes within internal cavities. The whole circulating system, besides, is usually in an irritable and excited condition; the pulse being considerably above the healthy standard, and the heart acting with unusual force and sharpness. Not unfrequently, a febrile condition at the same time exists; and when it does exist, it increases the intensity of the diathesis.

In the treatment of the hemorrhagic diathesis, Mr. M. directs attention to the following points:—1. *Energetic treatment at the outset,* for then only have we the blood favorable for coagulation, and the parts tolerant of pressure. 2. *The propriety of internal remedies*—astringents, sedatives, nauseants, and hydragogues—to obviate, if possible, the morbid condition of the blood; and administered either by the mouth or anus, according to circumstances. 3. *Abandonment of escharotics*—especially of the actual cautery, being at the best only occasionally and temporarily beneficial, and ultimately highly pernicious. 4. *Pressure,* preceded by a styptic, early, accurately, uniformly, and yet moderately applied, the best local means of treatment. 5. *Irritants and cupping,* at some distance from the bleeding point, not unlikely to prove beneficial; the former by creating an inflammation in a comparatively unimportant part, and thereby increasing the amount of fibrin in the general mass of blood; the latter by averting the sanguineous determination to the source of hemorrhage. 6. *Careful avoidance of simply febrile accession,* which would have the effect of exciting the circulation, at the same time diminishing still further the amount of fibrin. 7. *Patient persistence* in the foregoing system, without abrupt or frequent change of remedies. 8. In protracted cases, *nutritious, yet non-stimulating diet.* 9. Failing ordinary means, *transfusion* is to be attempted. 10. The question of *prophylaxis,* not irrational; the tendency being once known, its removal ought at least to be attempted.

